# Dietary Antioxidants, Quality of Nutrition and Cardiovascular Characteristics among Omnivores, Flexitarians and Vegetarians in Poland—The Results of Multicenter National Representative Survey WOBASZ

**DOI:** 10.3390/antiox12020222

**Published:** 2023-01-18

**Authors:** Magdalena Kwaśniewska, Małgorzata Pikala, Oliwia Grygorczuk, Anna Waśkiewicz, Urszula Stepaniak, Andrzej Pająk, Krystyna Kozakiewicz, Paweł Nadrowski, Tomasz Zdrojewski, Aleksandra Puch-Walczak, Andrzej Tykarski, Wojciech Drygas

**Affiliations:** 1 Department of Preventive Medicine, Medical University of Lodz, Żeligowskiego 7/9, 90-419 Lodz, Poland; 2 Department of Epidemiology and Biostatistics, Medical University of Lodz, 90-419 Lodz, Poland; 3 Department of Epidemiology, Cardiovascular Disease Prevention and Health Promotion, National Institute of Cardiology, 04-628 Warsaw, Poland; 4 Department of Epidemiology and Population Studies, Institute of Public Health, Jagiellonian University of Krakow, 31-007 Krakow, Poland; 5 Division of Cardiology and Structural Heart Diseases, Medical University of Silesia, 40-055 Katowice, Poland; 6 Department of Preventive Medicine and Education, Medical University of Gdansk, 80-210 Gdansk, Poland; 7 Department of Hypertensiology, Angiology and Internal Medicine, Poznan University of Medical Sciences, 61-701 Poznań, Poland

**Keywords:** antioxidants, dietary patterns, omnivores, flexitarian, vegetarian, cardiovascular, nutrition

## Abstract

Several reports have shown that more plant-based dietary patterns provide a higher intake of antioxidants compared to diets rich in meat and animal products. Data on the intake of key nutrients in cardiovascular disease (CVD) prevention in relation to particular diets in countries of Central and Eastern Europe are scarce. The aim of this study was to assess quality of nutrition and CVD characteristics in a representative sample of Polish adults following different dietary patterns. Special regard was paid to the intake of natural antioxidants. The study comprised 13,318 (7159 females) randomly selected adults aged ≥ 20 years participating in the National Multicentre Health Surveys WOBASZ and WOBASZ II. The subjects were categorized into groups of omnivores (92.4%), flexitarians (7.4%) and vegetarians (0.16%) according to type of diet using the Food Frequency Questionnaire and 24 h dietary recall. The obtained results showed that neither flexitarians nor vegetarians represented better dietary habits or lifestyle behaviors compared to omnivores. Flexitarians had significantly lower daily energy intake than omnivores, but their diet was rich in total fat (above 30% of daily energy consumption) and low in fiber. Omnivores declared a higher consumption of fresh vegetables (*p* < 0.001), fresh fruit (*p* < 0.01), coffee (*p* < 0.01) and tea (*p* < 0.05, in women only) than flexitarians. Omnivores had significantly higher intake of natural antioxidants (vitamin C, E, zinc in both genders and vitamin A in men) as compared with flexitarians. Among women, the highest adherence to the intake of recommended amounts of antioxidant nutrients was noted among omnivores. Among men, vegetarians had the highest proportion of meeting the guidelines for vitamin A (77.8%), E (66.7%) and C (66.7%), while the lowest proportions were found in flexitarians (69.9%, 39.5% and 32.4%, respectively). The groups did not differ in terms of smoking and physical activity level. There were no significant differences in the analyzed CVD characteristics between omnivores and flexitarians. In women, vegetarians had substantially lower BMI than omnivores (*p* < 0.05) and flexitarians (*p* < 0.05) and a lower mean serum glucose compared with omnivores (*p* < 0.01) and flexitarians (*p* < 0.05). Vegetarians had lower prevalence of hypertension and obesity than meat eaters. In conclusion, the results of the current research showed an inappropriate intake of several nutrients, including highly potent antioxidants, irrespective of the dietary regimen. Flexitarians did not have a more favorable CVD profile than omnivores. Taking into account the growing popularity of diets with reduced animal products, there is a need to elaborate strategies providing Polish adults with guidance regarding properly balanced nutrition.

## 1. Introduction

Dietary antioxidants are known for their multiple health benefits [1,2,3,4]. They exert an important role in the regulation of oxidative stress by maintaining the balance between the activity of free radicals and antioxidant potential [5,6]. Several studies have already shown that a diet rich in antioxidants, including certain vitamins, polyphenols and minerals, may decrease the risk of disease occurrence or prevent severe consequences by diminishing oxidative damage [1,2,3,4,5,6,7].

It should be, however, noted that among a variety of antioxidants there are dietary factors, which are not the key nutrients influencing human health. Available scientific data, including randomized controlled trials, show that beneficial cardiovascular effects are related with nutrition patterns as a whole rather than with specific nutrients alone. The exact mechanisms of cardioprotective diets (such as the Mediterranean diet) are uncertain. They are probably concerned with antioxidant and anti-inflammatory properties, improvement in endothelial function and lipid profile [8,9,10,11,12]. However, it is not obvious which component of the diet contributes to the observed health impact. There is also no consistency regarding the effects of supplemental intake of specific nutrients, including potent antioxidants. Several studies provided limited effects or even an adverse health impact of the supplemental use of beta-carotene, vitamin E, vitamin C and some trace minerals [11,12,13].

According to the various dietary guidelines, healthy and sustainable nutrition consists of diversity of plant-based foods which are a major source of the beneficial antioxidants. Health authorities recommend a diet beneficial for both health and the environment. They advocated on a diet which is rich in natural antioxidants through high intake of vegetables, fruits, pulses, whole grains and nuts, with moderate consumption of fish, eggs, poultry and dairy, and low intake of red meat, processed meat and fats, added sugar and sweets [14,15,16].

There is an increasing body of evidence that such a pattern is likely to meet the nutritional requirements as well as contribute to lower incidence and mortality due to chronic diseases, particularly CVD and metabolic disorders [3,4,7,17,18,19,20]. However, there is a need to control the quality of each type of diet, as unconsidered and not properly balanced diets may result in a deficiency of crucial nutrients, including highly potent antioxidants.

In many countries, dietary patterns have changed substantially over the last few decades. There is a visible trend of shifting towards diets that are rich in plant-based foods and low in red meat and highly-processed products [21]. However, while a strict plant-based diet is thought to be difficult to maintain, a flexitarian or semivegetarian approach seems to be a sustainable solution.

In the large study of Czech, Polish and Russian samples (the HAPIEE study), the Mediterranean dietary pattern was found to decrease the risk of death from all causes and CVD, while the typical Eastern European dietary pattern increased the risk of death from all causes and from CVD and cancer [22,23].

However, little is known for Eastern and Central Europe on diets that target the specific restriction of meat consumed. A systematic review of observational and intervention studies performed recently by Neufingerl and Eilander (2021) presented nutrient intakes in meat eaters and persons following plant-based diets [21]. Among 114 studies included in the analysis, all European statistics came from Western countries, mainly Finland, Switzerland, Germany and Great Britain. In Poland, quality of nutrition has already been assessed in national representative surveys, but so far, neither vegetarians nor flexitarians have been studied regarding particular nutrient intakes [24,25,26,27,28,29,30].

Several studies showed that sex/gender is one of the key factors in the assessment of cardiovascular health [31,32]. It is also important in the context of nutrition-related characteristics such as bioavailability and the absorption of several compounds [33]. The findings of Campesi et al. (2018) showed that plant-derived phenolic compounds might differently influence the health of male and female subjects [34]. The observed differences highlight the necessity of performing separate analyses for men and women.

Therefore, the aim of the study is to assess the quality of nutrition in relation to different types of dietary patterns in the adult population of Polish men and women. Special attention was paid to the intake of food groups containing natural antioxidants as well as selected nutrients with antioxidant properties. Moreover, the purpose of the study is to analyze sociodemographic and cardiometabolic characteristics of populations following different diet regimens using representative data of the Polish National Multicenter Health Survey (WOBASZ).

## 2. Material and Methods

### 2.1. Study Population and Data Selection

The study population was constituted of participants of the nationwide representative cross-sectional surveys WOBASZ (2003–2005) and WOBASZ II (2013–2014). Both surveys were carried out according to similar methodology. A detailed description of the WOBASZ surveys was published in previous publications [35,36]. Briefly, the surveys enrolled a total of 20,939 adults aged 20 years and older. The participants were randomly selected from the national population register. The sampling scheme covered the whole territory of Poland and was stratified according to gender, age, administrative units and type of urbanization. All the selected individuals received a personal invitation to the study. In both surveys, the measurements were performed by six academic centers in all 16 voivodships of Poland (108 measurement points in each survey).

The participants provided informed written consent. The study protocol was approved by the Ethical Committee of the National Institute of Cardiology in Warsaw (approval code IK-NP-0021-90/1175/09).

The study protocol included a questionnaire interview, anthropometric and blood pressure measurements and a blood sample collection. Data regarding socioeconomic factors, medical history, nutrition, smoking, physical activity, social support and depression were collected during the interview. The procedures were carried out by nurses and trained interviewers.

For the purpose of this analysis, only the records with complete data concerning daily food consumption were included. Therefore, the final sample comprised 13,318 persons aged 20 years and older (6159 males and 7159 females).

In the current research, the following sociodemographic measures were taken into account: age, educational level and marital status. Educational level was categorized as elementary (no education/primary school), secondary (high school vocational/incomplete high school/high school/vocational higher than high school) and university attainment (incomplete university/complete university education).

### 2.2. Assessment of Dietary Patterns and Nutrition

The study subjects were categorized according to their dietary patterns on the basis of the Food Frequency Questionnaire (FFQ) and a 24 h dietary recall.

The FFQ assessed usual consuming frequency of selected products over the last three months. There were 18 main groups of food products comprised in the questionnaire, including variety of meat products, fish, dairy, eggs, different fats, vegetables, fruit and beverages. For each item, the participants indicated the frequency at which it was consumed using one of the six possibilities (zero consumption, less frequently than once a week, once a week, two to three times a week, four to six times a week, everyday). The FFQ was used in order to estimate the frequency of the intake of red and white meat, meat products and fish, which was necessary to distinguish the groups of flexitarians, vegetarians and omnivores.

Daily food consumption was estimated by the means of a 24 h dietary recall. The respondents were asked to list all the meals, beverages and additional food products consumed within 24 h before the study. Food products were combined into groups according to their type and origin. Special attention was paid to the consumption of nutrients with potential antioxidant properties as well as dietary elements, which are crucial in nutrition guidelines provided by expert committees. The size of food portions was estimated using the photo albums of food provided by the National Food and Nutrition Institute in Warsaw [37]. Moreover, the usual supplementation was documented.

The study subjects were categorized according to the type of diet using the FFQ and 24 h recall. The three groups, i.e., vegetarians, flexitarians and omnivores, were determined based on the participants’ response to the questions concerning consumption of meat and other animal products. The participants were classified as vegetarians if they excluded meat (including red meat, poultry and fish), but consumed other animal products such as eggs, dairy and honey. Those who declared eating dairy products on regular basis but red meat or poultry at a frequency of ≥1 time per month but ≤1 time per week were classified as flexitarian [38]. Persons who consumed meat > 1 time per week were categorized as omnivores.

### 2.3. Assessment of Cardiovascular Characteristics

The analysis of smoking and physical activity level was based on self-reported data. People who had never smoked and exsmokers were included in the nonsmokers group. For the assessment of PA, participants were asked whether they took part in any regular physical activities (walking, jogging, swimming, bicycling, gardening, etc.) accumulating at least 30 min per session. Those who did were asked to recall the frequency of such activities, and 3 groups were created: high PA (4–7 times/week; moderate PA (2–3 times/week; low PA (less or no PA). Anthropometric (weight, height, waist circumference) and blood pressure measurements were carried by trained nurses using standardized methods. Body weight was measured without shoes and top garments, to the nearest 0.1 kg; height was assessed in the standing position without shoes, to the nearest 0.5 cm. Waist circumference was measured at the level of the umbilicus, using an inextensible measuring tape, to the nearest 0.5 cm.

Blood pressure was measured three times on the right arm after 5 min of rest in the seating position (automatic A & D UA-631 device approved by the Association for the Advancement of Medical Instrumentation). The average value achieved from the second and third measurements was used for the analysis. Fasting glucose and blood lipid concentrations were determined from frozen serum samples in the central laboratory (the Institute of Cardiology, Warsaw, Poland) using enzymatic methods and automated procedures.

Data regarding the presence of diabetes or hypertension and the medications taken were obtained through a questionnaire interview.

### 2.4. Statistical Analysis

Continuous variables were presented as means and standard deviations. Categorical variables were presented as counts and percentages. To compare the frequency and to assess the statistical significance of the categories of qualitative characteristics in the analyzed groups, the χ2 test was implemented. Given the sex differences in studied types of diet, we performed all the analyses separately for men and women. Statistical significance of the differences in the mean values of analyzed characteristics among the three studied groups was assessed using analysis of variance ANOVA and post hoc Tukey test. If conditions of the parametric ANOVA were not achieved, we used nonparametric Kruskal–Wallis analysis of variance and Dunn’s post hoc test. Logistic regression analysis was performed to identify dietary pattern that can contribute to selected clinical disorders. The results were shown as odds ratios (OR) with 95% confidence intervals (CI) and the analyses were adjusted for age and education.

The level of significance was considered to be at *p* value < 0.05. Statistical analyses were performed using STATISTICA for Windows XP, version 13.3 (StatSoft Polska, Kraków, Poland).

## 3. Results

A total of 13,318 subjects (53.7% women) were included in the analyses. Table 1 presents sociodemographic characteristics of the studied population according to the type of diet by gender. The groups differed significantly in terms of age, educational level (in men only) and marital status. Vegetarians were younger than flexitarians and omnivores in both genders, with a significantly lower mean age of vegetarians (36.3 years in women and 37.8 years in men) as compared to flexitarians (48.7 years in women; 50.3 years in men) and omnivores (47.5 years in women; 46.8 years in men) (*p* < 0.05). Most of the participants were middle-aged, in the age group 35–64 years. However, among women, most vegetarians were young adults below 35 years of age. More than 54% of women and two thirds of men had a secondary educational level. Compared with omnivores and flexitarians, elementary educational level was very rare among vegetarians. About one-third of vegetarians had a university attainment. Most omnivores and flexitarians were married, while among vegetarians there was a significantly higher proportion of singles, especially in women.

In order to assess the quality of nutrition and an adherence to current nutrition guidelines, the daily intake of macronutrients, cholesterol and fiber was estimated (Table 2) There were substantial differences in the daily intake of energy, macronutrients and cholesterol between the studied groups. Among women, omnivores declared a significantly higher intake of energy, water, total protein, animal protein, total fat, SFA, MUFA, PUFA and cholesterol in comparison with flexitarians (*p* < 0.001) and vegetarians (*p* < 0.05) for the intake of total protein, total fat and cholesterol; *p* < 0.01 for the intake of MUFA and PUFA; *p* < 0.001 for the intake of animal protein). The highest intake of plant proteins was noted among vegetarians (*p* < 0.001 for the comparison vs. flexitarians).

A similar distribution and level of significance was noted in some the analyzed characteristics among men. However, contrary to women, vegetarians did not have a lower intake of total fat compared with omnivores. Moreover, there were no significant differences in the consumption of SFA, MUFA, PUFA and cholesterol between vegetarians and other studied groups.

As compared with vegetarians, flexitarians declared significantly higher intake of animal proteins (*p* < 0.05 in both genders ) and cholesterol (*p* < 0.01 in women); and a lower intake of plant proteins (*p* < 0.05 in women). There were no differences between omnivores and flexitarians in the percent of energy intake from total fat, protein and carbohydrates in both genders. Among women, vegetarians had significantly lower % of energy from protein and fat than other studied groups (Table 2).

Table 3 presents the mean daily intake of products rich in antioxidants according to the dietary regimens of the study subjects. Significant differences were obtained only for the comparisons between omnivores and flexitarians. Among women, omnivores declared a higher consumption of fresh vegetables (*p* < 0.001), potatoes (*p* < 0.05), fresh fruit (*p* < 0.01), coffee (*p* < 0.01) and tea (*p* < 0.05) and a lower consumption of frozen vegetables (*p* < 0.05) than flexitarians. Vegetarians had higher intake of legumes, breakfast cereals and beer compared with other groups, but the differences were not statistically significant.

Among men, compared with flexitarians, omnivores declared a significantly higher intake of fresh vegetables (*p* < 0.01), potatoes (*p* < 0.01), fresh fruit (*p* < 0.05) and coffee (*p* < 0.05). Compared with other participants, vegetarians had a higher intake of fresh and frozen vegetables, fresh and dry fruit, nuts and tea, but the statistical analysis did not reveal significant differences.

The analysis of the daily intake of micronutrients, including natural antioxidants, for each dietary regimen is shown in Table 4. Among women, omnivores declared a significantly higher intake of most of the selected micronutrients, except for vitamin A (*p* < 0.001). Among men, significant differences were observed in the intake of all of the analyzed substances. Omnivores declared the highest consumption of most of the micronutrients, except for folates, with the highest intake in vegetarians.

Table 5 shows the proportion of subjects consuming recommended amounts of micronutrients, including antioxidants. The analysis revealed that in most cases, omnivores were closer to the recommendations than other participants of the study. However, among vegetarians, higher adherence was found in the intake of magnesium (in women), iron (in women), vitamin A and E (in men) and calcium and folate (in both genders). Importantly, none of the vegetarians reached the recommendations for vitamin D (in either gender) and potassium (in women).

The analysis of the intake of supplements showed that only a few percent of the omnivores and flexitarians declared regularly supplementing their diet. Among women, about 7% of omnivores and flexitarians regularly supplemented their diet with vitamin C and A and magnesium, about 3% declared taking vitamin B12 and calcium, while about 2% supplemented with zinc. Among men, the most popular supplements were also magnesium (3.%), vitamin C (3.7%) and vitamin A (2.8%). Similar patterns of taking particular supplements were observed in both omnivores and flexitarians. Among all vegetarians, only two men declared taking supplements, including vitamin B12 (data not shown in tables).

Table 6 shows that dietary regimen did not significantly influence the analyzed characteristics among men. In women, vegetarians had substantially lower BMI than omnivores (*p* < 0.05) and flexitarians (*p* < 0.05) and a lower mean serum glucose as compared with omnivores (*p* < 0.01) and flexitarians (*p* < 0.05). Among vegetarians, there were no cases of obesity in women or diabetes in both genders.

Additional analysis of the odds ratios of obesity, hypertension, hypercholesterolemia and diabetes adjusted for age and educational level did not reveal statistically significant differences between omnivores and flexitarians in most analyzed characteristics. Only the probability of hypertension in women was significantly higher among flexitarians than omnivores (OR 1.24; 95% CI 1.11–1.37). Among women, vegetarians had significantly lower odds of hypertension and hypercholesterolemia compared with omnivores (OR 0.73; 95% CI 0.53–0.93 and 0.81 95% CI 0.66–0.96, respectively) and hypertension compared with flexitarians (OR 0.45; 95% CI 0.22–0.68). Among men, vegetarians had significantly lower odds of hypertension and obesity compared with omnivores (OR 0.45; 95% CI 0.21–0.66 and OR 0.63; 95%CI 0.40–0.86, respectively) as well as compared with flexitarians (OR 0.16; 95% CI 0.08–0.24 and OR 0.42; 95% CI 0.20–0.62) (data not shown in Tables).

## 4. Discussion

The current analysis presents quality of nutrition and cardiovascular characteristics of Polish adults according to different dietary patterns in a national representative sample. Special regard was paid to the intake of natural antioxidants among omnivores, flexitarians and vegetarians. To our knowledge, this is the first larger study presenting the relationship between dietary regimens that target meat restriction, key nutrient intake and CVD outcomes performed in the Central-East of Europe.

There is growing evidence indicating that a reduction in meat consumption, especially red meat and processed meat, improves health and even contributes to lower mortality [14,18,19,20,21,33,34,35,36,37,38,39,40,41,42]. Nevertheless, an exclusion or a substantial reduction in animal products should be properly substituted in order to follow a well-balanced diet and ensure that nutritional requirements are met.

The results of the current research showed that plant-based diets were a very rare pattern in Poland (about 0.16%). Omnivores represented over 90% of the participants, while the prevalence of flexitarian diets reached 8.8% among women and 5.7% in men. We also performed an additional analysis in order to assess ten-year changes in the prevalence of dietary patterns in Poland. However, no significant differences were noted in terms of frequency of persons following a flexitarian or vegetarian diet (data not shown in this manuscript).

The prevalence of vegetarians in representative studies reported by other authors varies substantially, ranging from 0.5 to 4% in European societies [43,44,45,46]. The latest findings provided by Deliens et al. (2022) revealed that vegetarians and flexitarians represented 1.4% and 9.2% of Flemish adults, respectively [46]. Another set of data presenting the statistics of a Swiss urban population showed that about 1.2% of the studied sample represented vegetarians, while 15.6% declared a flexitarian dietary pattern [38]. A relatively high percentage of vegetarians and vegans participated in the French NutriNet-Santé study (over 3.4% of the 93,823 participants) [45].

In some societies, a visible shift from the omnivorous towards the flexitarian diet has been noted in recent years [38,43]. Such trends probably result from public health campaigns and experts’ recommendations increasing awareness of health benefits, as well as environmental sustainability connected with changing towards a more plant-based diet.

Poland, like other middle- and high-income countries, has a long tradition of a diet rich in animal-source food products. It is, however, probable that the prevalence of flexitarian or strict plant-based diets is growing. There are several reports indicating relatively high percentages of Polish vegetarians (reaching even 20–30%), but the estimates were driven by telephone or online surveys, in nonrepresentative populations, often performed by vegetarian associations [47,48]. The third edition of the WOBASZ survey, which is going to be carried out in the upcoming months, shall verify these assumptions.

It seems that a plant-based diet or a flexitarian regimen should be closer to the recommendations and abound in food rich in natural antioxidants. Surprisingly, our findings showed that neither flexitarians nor vegetarians represented better dietary habits compared to omnivores. Lower consumption of meat and animal products occurred unrelated to the intake of antioxidants and other key nutrients in the prevention of cardiac events. Flexitarians had significantly lower energy intake, but their diet was rich in fat (above 30% of daily energy consumption) and low in fiber. Both groups had comparable intakes of the main macronutrients calculated as a percentage of energy intake.

The analysis of the consumption of food products rich in natural antioxidants revealed that in the omnivore group, the intake of vegetables, fruits and coffee was significantly higher than among flexitarians. Significantly more omnivores met the recommendations concerning the minimum daily intake of vegetables, while comparing the intake of antioxidant vitamins and other micronutrients, omnivores declared higher consumption of most of the analyzed elements, especially vitamin C, vitamin E and zinc in both genders and vitamin A in men.

The vegetarian group was very limited, but some significant differences were obtained. Compared to other groups, female vegetarians’ diets were low in total fat, SFA and cholesterol, as well as total and animal proteins. Among men, the substantial difference was noted only in the intake of total and animal proteins. The intake of some nutrients among vegetarians was lower than in other groups, particularly vitamin D and B group vitamins.

Although our findings showed that the mean intake of most analyzed micronutrients was within the recommended values, the proportion of subjects consuming the required daily amounts of crucial nutrients was far from satisfying. Quite surprisingly, the prevalence of female vegetarians with the appropriate intake of antioxidant vitamins A, C and E was lower than among omnivores. Similar results were obtained for vitamins B6 and B12. The above findings are of particular interest in the context of CVD prevention, as vitamins A, C and E may reduce oxidative modification of LDL-C particles, while folate and vitamins B6 and B12 play a crucial role in the metabolism of homocysteine [49]. Other worrisome results concerned the intake of potassium, calcium, vitamin D and folate in all studied groups and zinc and niacin among vegetarians. Of note, none of the vegetarians consumed the recommended amounts of potassium (women), vitamin D or niacin (in either gender). To make matter worse, vegetarians generally did not take supplements in order to compensate for the potential deficiencies.

An unsatisfying quality of dietary habits among Polish adults has already been presented in previous papers [50,51,52,53,54,55]. Waśkiewicz et al. (2016) revealed that the diet of the Polish population falls short of the recommendations relevant for CVD prevention and health promotion [51]. The Healthy Diet Indicator score used in this study showed that a healthy diet was noted in only 15% of adult Poles, and most subjects were characterized by a low-quality diet. Nevertheless, most subjects believed that their diet was appropriate. However, in some urban populations, the proportion of people who follow a Mediterranean diet pattern can be surprisingly high, reaching about 30% [22]. A comparison of diet quality between the populations of Polish and Norwegian adults was provided by Janowska-Miasik et al. (2021) [25]. The authors found that the diet of Norwegians was better balanced in terms of food consumed and micronutrient intakes. Insufficient intake was found particularly for calcium, magnesium, potassium and thiamine. However, the consumption of folate and vitamin D by both genders and the intake of iron among women were inadequate in both countries. Other previous analyses performed within the frame of the WOBASZ and HAPIEE studies presented detailed data on dietary total antioxidant potential and polyphenol intake in the Polish population [24,26,28,50,51,52,53,54,55]. The main dietary sources of polyphenols and antioxidants in Polish adults were coffee and tea, and then vegetables and fruit. In the current research, we were interested if those who reduce or exclude animal products had a higher intake of food items rich in antioxidants. Unexpectedly, the findings showed that the highest consumption of the above-mentioned products was noted among omnivores. The obtained results are of particular importance, as it has been recently shown that a higher intake of polyphenols, antioxidant vitamins and minerals was associated with the dietary total antioxidant capacity and a reduced odds ratio for cardiovascular diseases in the Polish population [55].

Generally, little is known about the diet quality of flexitarians compared to omnivores or plant-based regimens. In the available literature, we did not find any scientific analyses concerning the prevalence and quality of a flexitarian diet in Poland. In the latest report by Bruns et al. (2022) performed among 94 middle-aged Germans, flexitarians had a higher-quality diet compared to omnivores, but lower than vegans [56]. Another comparative study showed that nutrient intake was substantially different regarding various dietary patterns [57]. The strongest differences in diet quality were found between omnivores and vegans, with flexitarians placed between meat eaters and vegetarians. The data from the NuEva screening showed that the reduced consumption of animal products in the following order: omnivores > flexitarians > vegetarians > vegans, is associated with a decreased intake of energy and fat, particularly SFA, cholesterol, disaccharides and total sugar, as well an increased intake of fibers and micronutrients, including those with antioxidant properties such as vitamin E, beta carotene and manganese [57]. Some critical nutrients were, however, identified for flexitarians with intakes higher than recommended, such as total fat, SFA, disaccharides and total sugar. The average intake of carbohydrates, particularly dietary fiber, n-3 PUFA, pantothenic acid, vitamin B12, vitamin D, vitamin E, iron (women), potassium and zinc were below the recommendations. Moreover, Clarys et al. (2014) showed that the diet quality and nutrient intake of semivegetarian and pescovegetarian diets were in-between the vegan and omnivorous values [58]. Subjects who ate meat less often than once a week had higher intake of food rich in natural antioxidants. Among omnivores, the percentage of subjects above the median intake was lower compared to less-meat- and plant-based diets for vegetables, legumes, fruit, nuts and cereals. The above findings are in line with several other studies investigating the relationship between diet regimen and diet quality. According to the meta-analysis by Parker et al. (2019), vegetarians or vegans had higher overall diet quality compared with nonvegetarians in 9 of 12 studies [59]. Vegetarians were substantially closer to experts’ recommendations for total fruit, whole grains, seafood and plant protein, as well as sodium intake.

As mentioned earlier, the quality of nutrition of the flexitarians in our study was unsatisfying and far from current dietary recommendations. The percentage of subjects consuming recommended amounts of micronutrients, including antioxidants, was lower than among omnivores. These findings indicate that Polish adults choosing a diet with reduced meat intake should have a greater nutritional knowledge in order to avoid potential deficiencies. This group of people should be of particular interest of public health specialists and dietitians, as the flexitarian or semivegetarian regimen is growing in popularity.

Contrary to flexitarians, there are much more data concerning nutrition quality among people following vegetarian diets [21,38,43,44,57]. The majority of studies show that well-balanced plant-based diets that are adequately supplemented can fulfill the requirements for antioxidants and other important nutrients. Compared with omnivores, vegetarians and vegans usually have lower dietary intakes of calcium, iodine, vitamin D and B12. The majority of previous studies’ authors documented better quality of diets among vegetarians and vegans compared to omnivores. Schupbach et al. (2017) noted significantly higher daily intakes of vitamin C, E, B1, B6 and folic acid among vegetarians compared with omnivores [43]. The highest intakes of those nutrients were found in vegans. Data on antioxidant intake and status provided recently by Dawczynski et al. (2022) indicated the highest cardioprotective potential of the vegan diet [57].

A systematic review of 141 studies performed by Neufingerl and Eilander (2021) showed that there were nutrient inadequacies across all dietary patterns, including vegan, vegetarian and meat-based diets [21]. The presented findings imply that plant-based dietary patterns can increase the risk of inadequate intake of certain nutrients, mainly present or more bioavailable in animal foods, but can improve the intake of nutrients abundant in plant foods (including antioxidant vitamins and minerals). Conversely, they provided the results that meat eaters were more at risk of an inadequate intake of fiber, PUFA, ALA, vitamin E, folate, magnesium and other nutrients that are more present in plant foods [15].

In our study, the results regarding micronutrient intake among vegetarians are generally not in line with the findings of the above-mentioned authors. The omnivores consumed the highest amounts of nutrients that are important in CVD prevention. However, similar to other studies [43,57], we found lower fat and cholesterol intake in vegetarians.

Other Polish studies investigating vegetarians’ attitudes and dietary knowledge revealed more optimistic results, especially regarding the awareness of the necessity of the proper composition of the diet and supplementation [47,60]. The majority of vegetarians declared supplementing vitamins D and B12. In the report of Marciniak et al. (2021), the nutritional knowledge of 390 studied vegetarians was satisfactory. They seemed to be aware of how to balance their diet and the nutrients that should be supplemented, even though a vast majority of them did not consult a dietitian [47]. However, it was not a representative study; the analysis was conducted among volunteers via Facebook using a computer-assisted web interviewing method.

Contrary to our expectations, the studied flexitarians and omnivores did not differ significantly in terms of analyzed cardiovascular characteristics. These findings are in contrast to other authors, reporting a more favorable cardiometabolic profile among individuals with reduced meat intake compared with omnivores [20,38,43,57]. In most studies, flexitarians and vegetarians had lower blood pressure and total and LDL-cholesterol concentration, as well as lower prevalence of hypercholesterolemia or diabetes [44,57]. The analysis performed among 10,797 inhabitants of Swiss urban areas in the years 2005–2017 showed that participants who adhered to any diet excluding or reducing meat intake had a better CVD profile, including lower BMI, total cholesterol and a prevalence of hypertension as compared with omnivores [38]. According to Dawczyński et al. (2022) flexitarians had a lower body weight, BMI and body fat percentage in comparison to omnivores. The authors concluded that flexitarian, vegetarian and vegan diets are nutrient-dense and could be recommended for weight management [57].

However, women who followed a vegetarian diet had significantly lower mean BMI and glucose concentration, as well as a probability of hypertension and obesity in comparison with meat eaters, confirming the findings of previous studies [20,38,43,44,57]. However, lower prevalence of hypercholesterolemia was observed only among women in the comparison of vegetarians and omnivores.

Due to low prevalence of the group following a vegetarian diet pattern, any conclusions should be drawn with caution.

The analysis of other healthy behaviors in our study did not provide substantial differences in physical activity level and prevalence of smoking according to dietary choices. More than half of the participants declared a sedentary lifestyle and about one-third regularly smoked cigarettes. Therefore, the present findings cannot confirm the assumption that flexitarians and vegetarians follow a healthier lifestyle. The obtained results to some extent confirm the findings of other authors, which were not consistent in terms of smoking statistics and physical activity level among groups following different diet regimens. The study of Wozniak et al. (2020) showed similar prevalence of current smokers among omnivores and flexitarians, but lower in pescatarians and vegetarians representing a Swiss urban population [38]. In contrast, no differences in terms of frequency of smoking were found in other studies with participants representing various dietary regimens [44,45,61]. Similar to our results, physical activity was not found to be significantly different between omnivores and those following plant-based diets in the report by Schüpbach et al. (2017) [43].

Previous analyses performed within the frame of WOBASZ and other large surveys in Poland showed high exposure to CVD risk factors, including an unsatisfactory level of physical activity and low prevalence of a healthy lifestyle of adults in Poland as a whole [53,54]. In addition, Gajda et al. (2022) recently provided results of the SCREEN-14 study (Seniors in the Community: Risk Evaluation for Eating and Nutrition) demonstrating that low physical activity level and smoking tobacco was associated with higher nutritional risk [62]. These findings are of particular importance, as besides proper dietary patterns, smoking and sedentary lifestyle are among the crucial factors contributing to CVD health outcomes [63,64].

Several studies indicated the importance of such factors as sex, age (including hormonal changes) or education in the assessment of CVD burden and lifestyle modifications. It seems that younger and better-educated persons are more health- and environment-conscious, as well as more willing to make changes in their lifestyle. In the present study, vegetarians were younger and had a higher educational level compared with other participants, which is in line with the findings of other authors [38,43,45]. In the current research, we did not perform a detailed analysis investigating the relationship between sociodemographic characteristics and quality of nutrition according to a dietary regimen. However, vegetarians who were about 10-12 years younger than other participants did not present a substantially more beneficial lifestyle, including dietary patterns, smoking and physical activity level.

The obtained results showed significant sex–gender differences in nutrition patterns, intake of most analyzed compounds and cardiovascular profile. It seems obvious that scientific research in this field should include separate analyses for males and females. Moreover, due to remarkable discrepancies in dietary choices and adherence to current recommendations among omnivores, flexitarians and vegetarians, there is a need for elaborating personalized diets. Consequently, further preventive and therapeutic approaches should be precisely tailored to the revealed differences in order to obtain an optimal effect.

It is also documented that ageing and menopausal status in women might induce several changes in cardiovascular status [65,66]. Due to hormonal alterations, particularly estrogen depletion, postmenopausal women are at increased risk of deficiencies in the nutritional status of certain nutrients [67]. There is also evidence that antioxidant status might be altered during the menopausal period, as well the bioavailability of certain nutrients [65,66]. A far as socioeconomic status is concerned, previous studies revealed that a higher socioeconomic status was significantly associated with better dietary habits among Polish adults, including a higher intake of dietary antioxidants [26,29,30].

It seems that health-oriented lifestyle, reliable nutritional knowledge and motivation are the key to follow a proper dietary pattern rich in highly potent nutrients. Even strict vegan diets may adhere to the current recommendations if they result from conscious decisions and thoughtful choices. Interesting findings provided by Wirnitzer et al. (2022) demonstrated that among marathon runners with different dietary regimens, vegans had a higher tendency toward the consumption of frequently recommended food groups in favor of health [68]. These results are in line with growing evidence that well-designed plant-based diets can fulfill the nutritional requirements considering health and a successful endurance performance [68,69,70].

Some limitations of the present study should be acknowledged. Most importantly, the sample of vegetarians was very low, which probably influenced the power issues. Moreover, the nutrient intake was assessed by means of a single 24 h recall, which could affect the results. However, a 24 h dietary recall is a common method used in large population studies. Importantly, unlike some previous studies, dietary patterns were not identified by web-based, self-administered 24 h dietary records, but by a method using a combined 24 h recall and an FFQ. If we assessed dietary patterns only by means of a 24 h recall, the numbers of vegetarians and flexitarians were several times higher.

Although the study protocol did not allow us to assess the duration of a specific diet regimen, the participants were asked whether their diet on a given day was typical of their usual nutrition in order to make the dietary recall more precise. Moreover, the analysis did not contain all of the potent dietary antioxidants, for example, carotenoids or polyphenols.

In order to eliminate the false findings resulting from multiple comparisons, the Bonferroni correction was used. The Bonferroni correction is a multiple-comparison correction used when several statistical tests are being performed simultaneously (since while a given alpha value alpha may be appropriate for each individual comparison, it is not for the set of all comparisons). In order to avoid a lot of spurious positives, the alpha value needs to be lowered to account for the number of comparisons being performed.

Further, due to the cross-sectional study design, we were not able to address a problem of causality between dietary regiments and CVD risk factors.

Importantly, all the procedures were carried out with the standardized methodology by nurses and trained interviewers, which is an important advantage in reference to studies using data collection from self-reported online or mailed questionnaires. Finally, the recruitment to the study was independent from any organizations with potential conflicts of interest, such as vegetarian associations or industries. The presented results are based on comprehensive multicenter national representative surveys, which provide objective and reliable data.

In conclusion, the results of the current research showed inappropriate intakes of several nutrients irrespective of the studied dietary regimen. Persons who excluded or reduced their consumption of meat did not have properly balanced diets, including the intake of highly potent antioxidants. Flexitarians did not have more favorable CVD profiles than regular meat eaters. The obtained results raise particular concerns in the context of unsatisfying cardiovascular profiles of all study participants, which highlights the necessity for the development of more effective public health interventions in this area. Due to remarkable sex differences in dietary choices, nutrition patterns and adherence to current recommendations, there is a need to elaborate on preventive and therapeutic dietary approaches tailored to the specific needs of males and females.

Taking into account current experts’ guidelines as well as the growing popularity of diets with reduced animal products, there is a need to elaborate strategies in order to provide Polish adults with guidance and facilitate behavior change to enable the best adherence to the dietary recommendations.

## Figures and Tables

**Table 1:** Sociodemographic characteristics of the studied sample according to the dietary pattern, by gender (National Multicenter Health Survey WOBASZ and WOBASZ II, *n* = 13 318).

Sociodemographic Characteristics	Women *n* = 7159				Men *n* = 6159			
	Omnivores *N* = 6515	Flexitarians *N* = 630	Vegetarians *N* = 12	*p* Value	Omnivores *N* = 5797	Flexitarians *N* = 352	Vegetarians *N* = 9	*p* Value
Age groups, *n* (%)								
<35 years	1630 (25.0)	155 (24.6) *	7 (58.3)	<0.001	1505 (26.0)	68 (19.3) *	3 (33.3)	<0.05
35–64 years	3856 (59.2)	336 (53.3) *	4 (33.3)		3399 (58.6)	221 (62.8) *	6 (66.7)	
>64 years	1029 (15.8)	139 (22.1) *	1 (8.3)		893 (15.4)	63 (17.9) *	0 (0.0)	
Educational level, *n* (%)								
Elementary	1413 (21.7) *	156 (24.8) *	2 (18.2)	>0.05	1061 (18.3) *	82 (23.4) *	0 (0.0)	<0.05
Secondary	3821 (58.7) *	343 (54.5) *	6 (54.5)		3863 (66.7) *	222 (63.4) *	6 (66.7)	
University	1272 (19.6) *	130 (20.7) *	3 (27.3)		867 (15.0) *	46 (13.2) *	3 (33.3)	
Marital status, *n* (%)								
Single	885 (13.6) *	115 (18.3) *	8 (72.7)	<0.001	1287 (22.2) *	73 (20.8) *	4 (44.4)	<0.05
Married	4444 (68.2) *	357 (56.8) *	3 (27.3)		4135 (71.4) *	242 (68.9) *	4 (44.4)	
Divorced	331 (5.1) *	58 (9.2) *	0 (0.0)		199 (3.4) *	20 (5.7) *	1 (11.1)	
Widowed	850 (13.1) *	99 (15.7) *	0 (0.0)		172 (3.0) *	16 (4.6) *	0 (0.0)	

Data are presented as number (percentage) of participants. * Statistically significant difference between women and men.

**Table 2:** Daily intake of energy, macronutrients, cholesterol and fiber among the study participants.

	Women			Men		
	Omnivores	Flexitarians	Vegetarians	Omnivores	Flexitarians	Vegetarians
Energy, kcal	1702.8 ± 665.9 ^a,^*	1480.9 ± 670.4 *	1532.1 ± 512.6	2413.5 ± 993.1 ^a,^*	2080.0 ± 879.8 *	2066.5 ± 989.9
Total protein, g	59.8 ± 24.7 ^a,b,^*	51.3 ± 24.1 *	41.9 ± 18.7	86.1 ± 37.7 ^a,b,^*	71.0 ± 30.2 *	51.5 ± 14.3
Protein (%)	14	13.9	10.9	14.3	13.7	10
Animal protein, g	38.0 ± 20.4 ^a,b,^*	31.4 ± 19.6 *	16.5 ± 10.7 ^c^	56.1 ± 31.1 ^a,b,^*	44.5 ± 24.9 *	20.6 ± 12.9 ^c^
Plant protein, g	21.8 ± 8.8 ^a,^*	20.0 ± 9.6 *	25.5 ± 10.6 ^c^	30.0 ± 12.7 ^a,^*	26.5 ± 11.5 *	30.9 ± 12.0
Carbohydrates, g	229.2 ± 92.6 ^a,^*	207.1 ± 99.4 *	254.6 ± 102.3	302.0 ± 125.0 ^a,^*	271.1 ± 119.1 *	296.1 ± 119.7
Total fat, g	67.4 ± 34.2 ^a,b,^*	55.8 ± 32.5 *	44.3 ± 30.3	101.1 ± 53.5 ^a,^*	82.1 ± 44.6 *	82.0 ± 63.8
Fat (%)	35.4 ^*^	33.9 *	26.0	37.7 *	35.5 *	35.6
SFA, g	25.4 ± 14.2 ^a,^*	21.3 ± 13.3 *	16.9 ± 11.5	37.2 ± 21.8 ^a,^*	30.8 ± 19.1 *	35.6 ± 33.0
MUFA, g	26.6 ± 14.9 ^a,b,^*	21.6 ± 14.1 *	14.1 ± 10.6 *	41.4 ± 23.8 ^a,^*	33.2 ± 19.3 *	30.3 ± 23.0 *
PUFA, g	10.3 ± 6.9 ^a,b,^*	8.5 ± 6.7 *	9.5 ± 9.2	14.9 ± 9.6 ^a,^*	12.0 ± 8.5 *	10.6 ± 6.8
Linoleic acid, g	8.7 ± 6.1 ^a,^*	7.1 ± 5.8 *	8.5 ± 8.5	12.5 ± 8.4 ^a,^*	10.0 ± 7.5 *	8.6 ± 5.5
α-linolenic acid, g	1.4 ± 1.1 ^a,^*	1.2 ± 1.1 *	1.1 ± 1.1	2.0 ± 1.5 ^a,^*	1.7 ± 1.3 *	1.9 ± 1.5
Cholesterol, mg	235.0 ± 155.0 ^a,b,^*	201.3 ± 153.0 *	90.3 ± 59.4 ^c,^*	350.5 ± 238.2 ^a,^*	286.0 ± 198.9 *	216.2 ± 223.1 *
Fiber, g	17.4 ± 7.6 ^a,^*	15.9 ± 8.2 *	17.4 ± 10.1	22.2 ± 9.7 ^a,^*	20.0 ± 9.4 *	23.2 ± 11.3

Abbreviations: SFA—saturated fatty acids; MUFA—monounsaturated fatty acids; PUFA—polyunsaturated fatty acids. Post hoc analysis: ^a^ statistically significant difference between omnivores and flexitarians; ^b^ statistically significant difference between omnivores and vegetarians; ^c^ statistically significant difference between flexitarians and vegetarians; * statistically significant difference between women and men.

**Table 3:** Daily intake of selected food items containing antioxidants according to the dietary patterns of the study participants.

	Women			Men		
	Omnivores	Flexitarians	Vegetarians	Omnivores	Flexitarians	Vegetarians
Fresh vegetables, g	181.0 ± 150.4 ^a,^*	161.8 ± 148.4	130.3 ± 86.0 *	202.1 ± 179.4 ^a,^*	171.3 ± 188.3	346.3 ± 342.1 *
Frozen vegetables, g	5.7 ± 39.3 ^a^	10.8 ± 56.8	0.0	4.4 ± 35.5	5.2 ± 43.1	5.6 ± 16.7
Legumes, g	3.0 ± 13.5*	3.5 ± 15.9	10.8 ± 18.0	5.2 ± 20.6 *	4.3 ± 18.0	0.0
Potatoes, g	221.8 ± 216.7 ^a,^*	182.0 ± 224.2 *	134.1 ± 166.9	314.7 ± 286.0 ^a,^*	283.7 ± 268.9 *	153.1 ± 208.7
Processed vegetables, g	24.2 ± 45.8 ^a,^*	18.4 ± 43.6 *	7.5 ± 15.6	34.4 ± 61.7 *	37.1 ± 71.4 *	20.2 ± 29.9
Breakfast cereals, g	4.7 ± 16.8 *	5.3 ± 17.6	8.3 ± 28.9	2.7 ± 14.6 *	4.0 ± 18.0	0.0
Dinner cereals, g	5.7 ± 45.7 *	3.5 ± 28.9	0.0	10.2 ± 71.5 *	4.3 ± 57.6	0.0
Fresh fruit, g	212.8 ± 227.6 ^a,^*	197.4 ± 245.0	188.6 ± 301.2	181.6 ± 233.6 ^a,^*	166.6 ± 225.4	242.6 ± 256.6
Frozen fruit, g	0.09 ± 2.9	0.00	0.67 ± 2.3	0.04 ± 1.78	0.57 ± 10.7	0.00
Dried fruit, g	0.44 ± 5.8	0.62 ± 7.9	0.00	0.28 ± 4.8	0.51 ± 6.9	8.89 ± 26.7
Juices, mL	48.1 ± 133.0	41.5 ± 138.8	45.8 ± 107.6	46.9 ± 152.4	37.4 ± 126.5	47.2 ± 103.4
Wine, mL	1.26 ± 16.1 *	0.56 ± 8.2 *	0.00	4.47 ± 59.3 *	10.0 ± 88.2 *	11.1 ± 33.3
Beer, mL	7.33 ± 65.2 *	7.26 ± 64.1 *	25.0 ± 86.6	75.59 ± 259.2 *	73.81 ± 245.5 *	55.56 ± 166.7
Tea, mL	336.9 ± 251.9 ^a,^*	305.8 ± 251.8 *	285.0 ± 278.5	375.0 ± 287.3 *	382.9 ± 304.0 *	434.4 ± 351.6
Coffee, mL	184.6 ± 183.4 ^a,^*	167.9 ± 196.8 *	183.3 ± 414.7	155.5 ± 188.3 ^a,^*	134.3 ± 194.9 *	118.9 ± 124.3
Nuts, g	1.28 ± 11.8	0.98 ± 7.8	0.25 ± 0.86	1.39 ± 12.1	1.31 ± 10.6	6.67 ± 20.0
Seeds, g	0.44 ± 4.9	0.34 ± 4.0	0.00	0.33 ± 5.6	0.34 ± 5.4	0.00
Groats, g	5.82 ± 17.4 *	6.01 ± 17.4	5.70 ± 12.2	6.58 ± 22.0 *	6.24 ± 21.9	9.50 ± 22.7
Wholemeal bread, g	22.78 ± 46.8	18.82 ± 42.0	22.50 ± 41.4	22.02 ± 60.1	21.00 ± 64.1	25.00 ± 49.7

Post hoc analysis: ^a^ statistically significant difference between omnivores and flexitarians; * statistically significant difference between women and men.

**Table 4:** Daily intake of selected micronutrients, including antioxidants, according to the dietary pattern.

	Women			Men		
	Omnivores	Flexitarians	Vegetarians	Omnivores	Flexitarians	Vegetarians
Potassium, mg	2826.6 ± 1120.6 ^a,^*	2464.3 ± 1164.2 *	2249.6 ± 1068.8	3533.4 ± 1442.2 ^a,^*	3088.4 ± 1295.5 *	2582.1 ± 1415.4
Phosphorus, mg	952.9 ± 388.2 ^a,^*	849.1 ± 385.2 *	835.8 ± 419.9	1292.4 ± 555.8 ^a,^*	1115.6 ± 462.3 *	916.2 ± 265.9
Magnesium, mg	232.3 ± 91.4 ^a,^*	206.8 ± 93.3 *	222.4 ± 87.9	298.5 ± 120.0 ^a,^ *	268.7 ± 113.8 *	257.2 ± 143.7
Iron, mg	9.3 ± 4.3 ^a,^*	8.2 ± 4.4 *	7.8 ± 3.3	12.8 ± 6.6 ^a,^*	10.7 ± 4.6 *	10.0 ± 5.0
Zinc, mg	8.0 ± 3.3 ^a,^*	6.9 ± 3.2 *	6.0 ± 2.4	11.6 ± 5.1 ^a,^*	9.6 ± 4.0 *	7.7 ± 2.7
Copper, mg	0.99 ± 0.4 ^a,^*	0.90 ± 0.4 *	0.95 ± 0.4	1.24 ± 0.5 ^a,^*	1.08 ± 0.5 *	1.06 ± 0.6
Manganese, mg	3.8 ± 1.8 ^a,^*	3.5 ± 1.8 *	3.7 ± 1.9	4.7 ± 2.2 ^a,^*	4.4 ± 2.3 *	5.0 ± 2.4
Vitamin A, µg	1001.4 ± 1773.7 *	1006.3 ± 1850.8	538.7 ± 451.0 *	1296.9 ± 2698.0 ^a,^*	916.6 ± 706.8	1113.1 ± 734.9 *
Vitamin C, mg	79.7 ± 75.2 ^a^	75.6 ± 82.2	66.4 ± 64.7	80.2 ± 78.3 ^a^	69.2 ± 68.7	142.4 ± 125.0
Vitamin E, mg	9.2 ± 5.7 ^a,^*	7.8 ± 5.4 *	8.4 ± 7.1	12.0 ± 7.6 ^a,^*	10.2 ± 7.1 *	13.0 ± 8.3
Vitamin D, µg	2.58 ± 3.3 ^a,b,^*	2.29 ± 3.3 *	1.03 ± 1.8 ^c^	4.13 ± 4.9 ^a,b,^*	3.57 ± 4.9 *	1.98 ± 2.2
Vitamin B1, mg	0.98 ± 0.5 ^a,^*	0.81 ± 0.4 *	0.75 ± 0.4	1.52 ± 0.8 ^a,^*	1.20 ± 0.6 *	0.83 ± 0.3
Vitamin B2, mg	1.27 ± 0.7 ^a,^*	1.17 ± 0.6 *	0.91 ± 0.5	1.67 ± 1.0 ^a,b,^*	1.43 ± 0.7 *	1.09 ± 0.4 ^c^
Niacin, mg	14.2 ± 7.5 ^a,b,^*	11.2 ± 6.7 *	7.1 ± 2.1 ^c^	21.0 ± 11.2 ^a,b,^*	16.4 ± 8.7 *	9.3 ± 3.9
Vitamin B6, mg	1.55 ± 0.7 ^a,b,^*	1.30 ± 0.7 *	1.02 ± 0.6	2.13 ± 0.9 ^a,^*	1.80 ± 0.8 *	1.33 ± 0.8
Folate, µg	220.5 ± 102.1 ^a,^*	203.0 ± 100.3 *	213.6 ± 115.4	277.8 ± 134.6 ^a,b,^*	248.7 ± 110.2 *	287.8 ± 144.2
Vitamin B12, µg	3.25 ± 6.7 ^a,b,^*	2.91 ± 4.6 *	1.18 ± 1.0 ^c^	5.13 ± 13.1 ^a,^*	4.07 ± 9.7 *	1.55 ± 0.8

Data presented as means ± SD. Post hoc analysis: ^a^ statistically significant difference between omnivores and flexitarians; ^b^ statistically significant difference between omnivores and vegetarians; ^c^ statistically significant difference between flexitarians and vegetarians; * statistically significant difference between women and men.

**Table 5:** Proportion of subjects consuming recommended amounts of micronutrients, including antioxidants, by diet groups.

Micronutrients	Recommended Dietary Intake (Men/Women)	Proportion of Subjects Consuming Recommended Amounts, %							
		Women				Men			
		Omnivores	Flexitarians	Vegetarians	*p* Value	Omnivores	Flexitarians	Vegetarians	*p* Value
Potassium, mg	≥4700	5.7 *	3.3 *	0.0	<0.05	18.8 *	12.2 *	11.1	<0.01
Calcium, mg	19–50 years ≥ 800; above 50 years ≥ 1000	10.9 *	11.9 *	25.0	>0.05	17.8 *	16.8 *	33.3	>0.05
Phosphorus, mg	≥700	73.2 *	60.8 *	50.0	<0.001	89.3 *	82.1 *	88.9	<0.001
Magnesium, mg	≥350/265	30.6 *	23.2	41.7	<0.001	28.0 *	21.0	22.2	<0.05
Iron, mg	≥18/10	33.3 *	26.0 *	41.7	<0.001	12.8 *	6.8 *	11.1	<0.01
Zinc, mg	≥11/8	43.0 *	30.0	25.0	<0.001	48.1 *	31.5	11.1	<0.001
Copper, mg	≥0.7	76.1 *	63.2 *	66.7	<0.001	88.1 *	80.1 *	66.7	<0.001
Manganese, mg	2.3/1.8	92.1	85.1	83.3	<0.001	91.2	85.8	88.9	<0.01
Vitamin A, µg	≥630/500	74.8	70.2	50.0	<0.01	74.1	69.9	77.8	>0.05
Vitamin E, mg	≥10/8	50.0 *	37.5	41.7	<0.001	52.4 *	39.5	66.7	<0.001
Vitamin B1, mg	≥1.1/0.9	48.6 *	34.4 *	50.0	<0.001	66.0 *	50.3 *	11.1	<0.001
Vitamin B2, mg	≥1.1/0.9	71.5 *	62.4	41.7	<0.001	74.7 *	64.2	44.4	<0.001
Niacin, mg	≥16/14	42.1 *	25.6 *	0.0	<0.001	63.0 *	43.7 *	0.0	<0.001
Vitamin B6, mg	≥1.1/1.3	60.5 *	45.2 *	33.3	<0.001	88.4 *	82.4 *	44.4	<0.001
Vitamin C, mg	≥75/60	48.3 *	44.3 *	41.7	>0.05	38.3 *	32.4 *	66.7	>0.05
Folate, µg	≥320	12.6 *	11.3 *	16.7	>0.05	29.8 *	21.3 *	33.3	<0.01
Vitamin B12, µg	≥2	50.2 *	45.9 *	25.0	>0.05	68.5 *	63.1 *	33.3	<0.01
Vitamin D, µg	≥10	2.5 *	2.9 *	0.0	>0.05	6.1 *	6.0 *	0.0	>0.05

* Statistically significant difference between women and men.

**Table 6:** Association between dietary pattern and cardiovascular characteristics in the studied groups.

Cardiovascular Characteristics	Women			Men		
	Omnivores	Flexitarians	Vegetarians	Omnivores	Flexitarians	Vegetarians
Systolic blood pressure, mm Hg	128.78 ± 20.8 *	130.49 ± 22.3 *	129.08 ± 18.7	136.15 ± 18.6 *	138.93 ± 20.9 *	129.0 ± 10.8
Diastolic blood pressure, mm Hg	80.38 ± 11.4 *	81.34 ± 11.9 *	82.25 ± 10.0	82.82 ± 11.4 *	83.30 ± 12.3 *	81.06 ± 8.8
Current smoking, *n* (%)	1399 (21.5) *	146 (23.3) *	4 (36.4)	2005 (34.6) *	128 (36.6) *	1 (11.1)
Physical activity, *n* (%)						
4–7 times/week	1975 (30.3) *	184 (29.2)	1 (8.3) *	1934 (33.4) *	119 (33.8)	4 (44.4) *
2–3 times/week	886 (13.6) *	90 (14.3)	1 (8.3) *	824 (14.2) *	38 (10.8)	3 (33.3) *
Less or no PA	3654 (56.1) *	356 (56.5)	10 (83.4) *	3039 (52.4) *	195 (55.4)	2 (22.3) *
Body mass index, kg/m^2^	26.5 ± 5.7 *	26.6 ± 5.7	21.9 ± 3.1 ^a,b^	26.9 ± 4.5 *	26.8 ± 4.9	25.2 ± 4.5
Waist circumference, cm	86.8 ± 14.1 *	87.1 ± 14.3 *	76.0 ± 5.9 ^a,b,^*	96.0 ± 12.3 *	96.2 ± 12.7 *	91.3 ± 9.8 *
Glucose, mmol/L	5.13 ± 1.4 *	5.06 ± 1.3 *	4.29 ± 0.5 ^a,b^	5.35 ± 1.5 *	5.32 ± 1.3 *	4.77 ± 0.5
Total cholesterol, mmol/L	5.32 ± 1.2	5.27 ± 1.2	4.65 ± 1.5	5.32 ± 1.2	5.33 ± 1.3	5.32 ± 1.2
LDL-C, mmol/L	3.17 ± 1.0 *	3.12 ± 1.1	2.68 ± 1.2	3.21 ± 1.0 *	3.22 ± 1.1	3.05 ± 1.0
HDL-C, mmol/L	1.54 ± 0.4 *	1.56 ± 0.4 *	1.47 ± 0.5	1.35 ± 0.5 *	1.36 ± 0.5 *	1.37 ± 0.4
Triglycerides, mmol/L	1.35 ± 0.9 *	1.28 ± 0.6 *	1.08 ± 0.3 *	1.71 ± 1.6 *	1.63 ± 1.1 *	1.94 ± 1.1 *
Obesity, *n* (%)	1560 (23.9) *	162 (25.7)	0	1244 (21.5) *	79 (22.4)	1 (11.1)
Hypertension, *n* (%)	1725 (26.5) *	189 (30.0) *	2 (16.7)	2113 (36.5) *	151 (42.9) *	1 (11.1)
Hypercholesterolemia, *n* (%)	3384 (51.9) *	316 (50.2)	5 (41.7)	3118 (53.8) *	187 (53.1)	5 (55.5)
Diabetes, *n* (%)	465 (7.1) *	41 (6.5)	0	471 (8.1) *	27 (7.7)	0 (0.0)

Data presented as means ± SD unless otherwise stated. Abbreviations: LDL-C—low-density lipoproteins; HDL-C—high-density lipoproteins. Post hoc analysis: ^a^ statistically significant difference between omnivores and vegetarians; ^b^ statistically significant difference between flexitarians and vegetarians; * statistically significant difference between women and men.

## Data Availability

The data presented in this study are available in the article.

## References

[1] Baroni L., Sarni A., Zuliani C. (2021). Plant Foods Rich in Antioxidants and Human Cognition: A Systematic Review. Antioxidants.

[2] Rychter A.M., Hryhorowicz S., Słomski R., Dobrowolska A., Krela-Kaźmierczak I. (2022). Antioxidant effects of vitamin E and risk of cardiovascular disease in women with obesity—A narrative review. Clin. Nutr..

[3] Rana A., Samtiya M., Dhewa T., Mishra V., Aluko R.E. (2022). Health benefits of polyphenols: A concise review. J. Food Biochem..

[4] Islam S., Ahmed M., Ahsan H., Lee Y.-S. (2021). Recent Molecular Mechanisms and Beneficial Effects of Phytochemicals and Plant-Based Whole Foods in Reducing LDL-C and Preventing Cardiovascular Disease. Antioxidants.

[5] Halliwell B. (2007). Biochemistry of oxidative stress. Biochem. Soc. Trans..

[6] Petrucci G., Rizzi A., Hatem D., Tosti G., Rocca B., Pitocco D. (2022). Role of Oxidative Stress in the Pathogenesis of Atherothrombotic Diseases. Antioxidants.

[7] Szczepańska E., Białek-Dratwa A., Janota B., Kowalski O. (2022). Dietary Therapy in Prevention of Cardiovascular Disease (CVD)—Tradition or Modernity? A Review of the Latest Approaches to Nutrition in CVD. Nutrients.

[8] Lăcătușu C.-M., Grigorescu E.-D., Floria M., Onofriescu A., Mihai B.-M. (2019). The Mediterranean Diet: From an Environment-Driven Food Culture to an Emerging Medical Prescription. Int. J. Environ. Res. Public Health.

[9] Man A.W.C., Li H., Xia N. (2020). Impact of Lifestyles (Diet and Exercise) on Vascular Health: Oxidative Stress and Endothelial Function. Oxidative Med. Cell. Longev..

[10] Richardson L.A., Izuora K., Basu A. (2022). Mediterranean Diet and Its Association with Cardiovascular Disease Risk Factors: A Scoping Review. Int. J. Environ. Res. Public Health.

[11] Ashor A.W., Brown R., Keenan P.D., Willis N.D., Siervo M., Mathers J.C. (2018). Limited evidence for a beneficial effect of vitamin C supplementation on biomarkers of cardiovascular diseases: An umbrella review of systematic reviews and meta-analyses. Nutr. Res..

[12] A Jenkins D.J., Kitts D., Giovannucci E.L., Sahye-Pudaruth S., Paquette M., Mejia S.B., Patel D., Kavanagh M., Tsirakis T., Kendall C.W.C. (2020). Selenium, antioxidants, cardiovascular disease, and all-cause mortality: A systematic review and meta-analysis of randomized controlled trials. Am. J. Clin. Nutr..

[13] Mohammadifard N., Humphries K.H., Gotay C., Mena-Sánchez G., Salas-Salvadó J., Esmaillzadeh A., Ignaszewski A., Sarrafzadegan N. (2017). Trace minerals intake: Risks and benefits for cardiovascular health. Crit. Rev. Food Sci. Nutr..

[14] Willett W., Rockström J., Loken B., Springmann M., Lang T., Vermeulen S., Garnett T., Tilman D., DeClerck F., Wood A. (2019). Food in the Anthropocene: The EAT–Lancet Commission on healthy diets from sustainable food systems. Lancet.

[15] FAO/WHO (2019). Sustainable Healthy Diets—Guiding Principles.

[16] Visseren F.L.J., Mach F., Smulders Y.M., Carballo D., Koskinas K.C., Bäck M., Benetos A., Biffi A., Boavida J.-M., Capodanno D. (2021). ESC Guidelines on cardiovascular disease prevention in clinical practice. Eur. Heart J..

[17] Sofi F., Abbate R., Gensini G.F., Casini A. (2010). Accruing evidence on benefits of adherence to the Mediterranean diet on health: An updated systematic review and meta-analysis. Am. J. Clin. Nutr..

[18] Ibsen D.B., Christiansen A.H., Olsen A., Tjønneland A., Overvad K., Wolk A., Mortensen J.K., Dahm C.C. (2022). Adherence to the EAT-Lancet Diet and Risk of Stroke and Stroke Subtypes: A Cohort Study. Stroke.

[19] Xu C., Cao Z., Yang H., Hou Y., Wang X., Wang Y. (2022). Association Between the EAT-Lancet Diet Pattern and Risk of Type 2 Diabetes: A Prospective Cohort Study. Front. Nutr..

[20] Dinu M., Abbate R., Gensini G.F., Casini A., Sofi F. (2017). Vegetarian, vegan diets and multiple health outcomes: A systematic review with meta-analysis of observational studies. Crit. Rev. Food Sci. Nutr..

[21] Neufingerl N., Eilander A. (2021). Nutrient Intake and Status in Adults Consuming Plant-Based Diets Compared to Meat-Eaters: A Systematic Review. Nutrients.

[22] Stefler D., Malyutina S., Kubinova R., Pająk A., Peasey A., Pikhart H., Brunner E.J., Bobak M. (2017). Mediterra-nean diet score and total and cardiovascular mortality in Eastern Europe: The HAPIEE study. Eur. J. Nutr..

[23] Stefler D., Brett D., Sarkadi-Nagy E., Kopczynska E., Detchev S., Bati A., Scrob M., Koenker D., Aleksov B., Douarin E. (2020). Traditional Eastern European diet and mortality: Prospective evidence from the HAPIEE study. Eur. J. Nutr..

[24] Grosso G., Stepaniak U., Topor-Mądry R., Szafraniec K., Pająk A. (2014). Estimated dietary intake and major food sources of polyphenols in the Polish arm of the HAPIEE study. Nutrition.

[25] Janowska-Miasik E., Waśkiewicz A., Witkowska A.M., Drygas W., Markhus M.W., Zujko M.E., Kjellevold M. (2021). Diet quality in the population of Norway and Poland: Differences in the availability and consumption of food considering national nutrition guidelines and food market. BMC Public Health.

[26] Zujko M.E., Witkowska A.M., Waskiewicz A., Mironczuk-Chodakowska I. (2015). Dietary Antioxidant and Flavonoid Intakes Are Reduced in the Elderly. Oxid. Med. Cell. Longev..

[27] Grosso G., Stepaniak U., Micek A., Topor-Mądry R., Stefler D., Szafraniec K., Bobak M., Pająk A. (2015). A Med-iterranean-type diet is associated with better metabolic profile in urban Polish adults: Results from the HAPIEE study. Metabolism.

[28] Boylan S., Lallukka T., Lahelma E., Pikhart H., Malyutina S., Pajak A., Kubinova R., Bragina O., Stepaniak U., Gillis-Januszewska A. (2010). Socio-economic circumstances and food habits in Eastern, Central and Western European populations. Public Health Nutr..

[29] Bojar I., Owoc A., Humeniuk E., Wierzba W., Fronczak A. (2012). Inappropriate consumption of vitamins and minerals by pregnant women in Poland. Ann. Agric. Environ. Med..

[30] Zujko M.E., Waśkiewicz A., Drygas W., Cicha-Mikołajczyk A., Zujko K., Szcześniewska D., Kozakiewicz K., Witkowska A.M. (2020). Dietary Habits and Dietary Antioxidant Intake Are Related to Socioeconomic Status in Polish Adults: A Nationwide Study. Nutrients.

[31] Ruijter H.M.D., Haitjema S., Asselbergs F.W., Pasterkamp G. (2015). Sex matters to the heart: A special issue dedicated to the impact of sex related differences of cardiovascular diseases. Atherosclerosis.

[32] Kouvari M., Panagiotakos D.B., Chrysohoou C., Georgousopoulou E., Notara V., Tousoulis D., Pitsavos C., ATTICA & GREECS Studies Investigators (2019). Gender-specific, Lifestyle-related Factors and 10-year Cardiovascular Disease Risk; the ATTICA and GREECS Cohort Studies. Curr. Vasc. Pharmacol..

[33] Franconi F., Campesi I., Romani A. (2020). Is Extra Virgin Olive Oil an Ally for Women’s and Men’s Cardiovascu-lar Health?. Cardiovasc. Ther..

[34] Campesi I., Marino M., Cipolletti M., Romani A., Franconi F. (2018). Put “gender glasses” on the effects of phenolic compounds on cardiovascular function and diseases. Eur. J. Nutr..

[35] Broda G., Rywik S. (2005). Multicenter national Polish population health status tests—WOBASZ project with defined problems and treatment goals. Kardiol. Pol..

[36] Drygas W., Niklas A.A., Piwońska A., Piotrowski W., Flotyńska A., Kwaśniewska M., Nadrowski P., Puch-Walczak A., Szafraniec K., Bielecki W. (2016). Multi-centre National Population Health Examination Survey (WOBASZ II study): Assumptions, methods, and implementation. Pol. Heart J..

[37] Szponar L., Wolnicka K., Rychlik E. (2000). Album of Photographs of Food Products and Dishes.

[38] Wozniak H., Larpin C., De Mestral C., Guessous I., Reny J.-L., Stringhini S. (2020). Vegetarian, pescatarian and flexitarian diets: Sociodemographic determinants and association with cardiovascular risk factors in a Swiss urban population. Br. J. Nutr..

[39] Rocha J.P., Laster J., Parag B., Shah N.U. (2019). Multiple Health Benefits and Minimal Risks Associated with Vegetarian Diets. Curr. Nutr. Rep..

[40] Appleby P.N., Davey G.K., Key T.J. (2002). Hypertension and blood pressure among meat eaters, fish eaters, vegetarians and vegans in EPIC–Oxford. Public Health Nutr..

[41] Oussalah A., Levy J., Berthezène C., Alpers D.H., Guéant J.-L. (2020). Health outcomes associated with vegetarian diets: An umbrella review of systematic reviews and meta-analyses. Clin. Nutr..

[42] Kahleova H., Levin S., Barnard N.D. (2018). Vegetarian Dietary Patterns and Cardiovascular Disease. Prog. Cardiovasc. Dis..

[43] Schüpbach R., Wegmuller R., Berguerand C., Bui M., Herteraeberli I. (2017). Micronutrient status and intake in omnivores, vegetarians and vegans in Switzerland. Eur. J. Nutr..

[44] Elorinne A.-L., Alfthan G., Erlund I., Kivimäki H., Paju A., Salminen I., Turpeinen U., Voutilainen S., Laakso J. (2016). Food and Nutrient Intake and Nutritional Status of Finnish Vegans and Non-Vegetarians. PLoS ONE.

[45] Allès B., Baudry J., Méjean C., Touvier M., Péneau S., Hercberg S., Kesse-Guyot E. (2017). Comparison of Sociodemographic and Nutritional Characteristics between Self-Reported Vegetarians, Vegans, and Meat-Eaters from the NutriNet-Santé Study. Nutrients.

[46] Deliens T., Mullie P., Clarys P. (2021). Plant-based dietary patterns in Flemish adults: A 10-year trend analysis. Eur. J. Nutr..

[47] Marciniak S., Lange E., Laskowski W. (2021). Assessment of the knowledge of nutritional recommendations and way of nutrition in vegetarians and vegans. Rocz Panstw Zakl Hig..

[48] Skorek P., Glibowski P., Banach K. (2019). Nutrition of vegetarians in Poland—A review of research. Rocz Panstw Zakl Hig..

[49] Dhonukshe-Rutten R.A., de Vries J.H., de Bree A., van der Put N., van Staveren W.A., de Groot L.C. (2009). Dietary intake and status of folate and vitamin B12 and their association with homocysteine and cardio-vascular disease in European populations. Eur. J. Clin. Nutr..

[50] Zujko M., Witkowska A., Waśkiewicz A., Sygnowska E. (2012). Estimation of dietary intake and patterns of polyphenol consumption in Polish adult population. Adv. Med. Sci..

[51] Waśkiewicz A., Szcześniewska D., Szostak-Węgierek D., Kwaśniewska M., Pająk A., Stepaniak U., Kozakiewicz K., Tykarski A., Zdrojewski T., Zujko M.E. (2016). Are dietary habits of the Polish population consistent with the recommendations for prevention of cardiovascular disease?—WOBASZ II project. Kardiol. Pol..

[52] Waśkiewicz A., Zujko M.E., Szcześniewska D., Tykarski A., Kwaśniewska M., Drygas W., Witkowska A.M. (2019). Polyphenols and dietary antioxidant potential, and their relationship with arterial hypertension: A cross-sectional study of theadult population in Poland (WOBASZ II). Adv. Clin. Exp. Med..

[53] Kwaśniewska M., Pikala M., Aranowska A., Bielecki W., Kozakiewicz K., Pająk A., Tykarski A., Zdrojewski T., Nadrowski P., Piwoński J. (2021). Ten-year changes in adherence to a healthy lifestyle: The results of the WOBASZ Surveys. Pol. Arch. Intern. Med..

[54] Pająk A., Jankowski P., Zdrojewski T. (2022). The burden of cardiovascular disease risk factors: A current problem. Kardiol. Pol..

[55] Zujko M.E., Waśkiewicz A., Witkowska A.M., Cicha-Mikołajczyk A., Zujko K., Drygas W. (2022). Dietary Total Antioxidant Capacity—A New Indicator of Healthy Diet Quality in Cardiovascular Diseases: A Polish Cross-Sectional Study. Nutrients.

[56] Bruns A., Mueller M., Schneider I., Hahn A. (2022). Application of a Modified Healthy Eating Index (HEI-Flex) to Compare the Diet Quality of Flexitarians, Vegans and Omnivores in Germany. Nutrients.

[57] Dawczynski C., Weidauer T., Richert C., Schlattmann P., Dawczynski K., Kiehntopf M. (2022). Nutrient Intake and Nutrition Status in Vegetarians and Vegans in Comparison to Omnivores—the Nutritional Evaluation (NuEva) Study. Front. Nutr..

[58] Clarys P., Deliens T., Huybrechts I., Deriemaeker P., Vanaelst B., De Keyzer W., Hebbelinck M., Mullie P. (2014). Comparison of Nutritional Quality of the Vegan, Vegetarian, Semi-Vegetarian, Pesco-Vegetarian and Omnivorous Diet. Nutrients.

[59] Parker H.W., Vadiveloo M.K. (2019). Diet quality of vegetarian diets compared with nonvegetarian diets: A systematic review. Nutr. Rev..

[60] Grzelak T., Suliga K., Pełczyńska M., Sperling M., Czyżewska K. (2017). Ocena częstości stosowania suplementów diety wśród wegetarian oraz osób odżywiających się tradycyjnie. Probl. Hig. Epidemiol..

[61] Wirnitzer K., Boldt P., Lechleitner C., Wirnitzer G., Leitzmann C., Rosemann T., Knechtle B. (2018). Health Status of Female and Male Vegetarian and Vegan Endurance Runners Compared to Omnivores—Results from the NURMI Study (Step 2). Nutrients.

[62] Gajda R., Raczkowska E., Mazurkiewicz D., Suliga E. (2022). Differentiation of Nutritional Risk among Polish Seniors Based on Selected Lifestyle Characteristics. Nutrients.

[63] Gąska I., Sygit K.M., Cipora E., Sygit M., Krakowiak J. (2020). Factors determining health behaviours of the 50+ population with cardiovascular diseases. Ann. Agric. Environ. Med..

[64] Sygit K.M., Sygit M., Wojtyła-Buciora P., Lubiniec O., Stelmach W., Krakowiak J. (2019). Physical activity as an important element in organizing and managing the lifestyle of populations in urban and rural environments. Ann. Agric. Environ. Med..

[65] Campesi I., Occhioni S., Tonolo G., Cherchi S., Basili S., Carru C., Zinellu A., Franconi F. (2016). Age-ing/Menopausal Status in Healthy Women and Ageing in Healthy Men Differently Affect Cardiometabolic Parameters. Int. J. Med. Sci..

[66] Yang S., Feskanich D., Willett W.C., Eliassen A.H., Wu T. (2014). Association between global biomarkers of oxidative stress and hip fracture in postmenopausal women: A prospective study. J. Bone Miner. Res..

[67] Nasiadek M., Stragierowicz J., Klimczak M., Kilanowicz A. (2020). The Role of Zinc in Selected Female Reproductive System Disorders. Nutrients.

[68] Wirnitzer K., Wagner K.-H., Motevalli M., Tanous D., Wirnitzer G., Leitzmann C., Rosemann T., Knechtle B. (2022). Dietary Intake of Vegan and Non-Vegan Endurance Runners—Results from the NURMI Study (Step 2). Nutrients.

[69] Król W., Price S., Śliż D., Parol D., Konopka M., Mamcarz A., Wełnicki M., Braksator W. (2020). A Vegan Athlete’s Heart—Is It Different? Morphology and Function in Echocardiography. Diagnostics.

[70] Torna E., Smith E., Lamothe M., Langkamp-Henken B., Andrade J.M. (2021). Comparison of diet quality of US adults based on primary motivation for following a vegetarian diet: A cross-sectional online study. Nutr. Res..

